# Nomogram prediction of overall survival based on log odds of positive lymph nodes for patients with penile squamous cell carcinoma

**DOI:** 10.1002/cam4.3232

**Published:** 2020-06-10

**Authors:** Wenwen Zheng, Kangqi Li, Weiwei Zhu, Yuexia Ding, Qingna Wu, Qiling Tang, Congxiao Lu, Quan Zhao, Shengqiang Yu, Chenyu Guo

**Affiliations:** ^1^ Department of Education Yantai Yuhuangding Hospital Qingdao University Yantai China; ^2^ Drug Clinical Trial Agency Yantai Yuhuangding Hospital Qingdao University Yantai China; ^3^ Department of Pharmacy Yantai Yuhuangding Hospital Qingdao University Yantai China; ^4^ Department of Urology Yantai Yuhuangding Hospital Qingdao University Yantai China

**Keywords:** LODDS, nomogram, overall survival, penile squamous cell carcinoma, SEER

## Abstract

**Purpose:**

This study aimed to establish a nomogram to predict the long‐term overall survival (OS) for patients with penile squamous cell carcinoma (PSCC).

**Method:**

The PSCC patients receiving regional lymph node dissection (RLND) were enrolled from the Surveillance, Epidemiology, and End Results (SEER) database between 2004 and 2015. The dataset of all eligible patients were used to develop the predictive model. The significant independent predictors were identified through Cox regression modeling based on the Bayesian information criterion and then incorporated into a nomogram to predicted 1‐, 3‐, and 5‐year OS. Internal validation was performed using the bootstrap resampling method. The model performance was evaluated using Harrell's concordance index (C‐index), calibration plots, integrated discrimination improvement (IDI), net reclassification improvement (NRI), and decision curve analysis (DCA).

**Results:**

Totally, 384 eligible PSCC patients were enrolled from the SEER database. A nomogram for OS prediction was developed, in which three clinical variables significantly associated with OS were integrated, including age, N classification, and log odds of positive lymph nodes (LODDS). The C‐index of the nomogram (0.746, 95% CI: 0.702‐0.790) was significantly higher than that of the American Joint Committee on Cancer (AJCC) staging system (0.692, 95% CI: 0.646‐0.738, *P* = .020). The bootstrap optimism‐corrected C‐index for the nomogram was 0.739 (95% CI: 0.690‐0.784). The bias‐corrected calibration plots showed the predicted risks were in good accordance with the actual risks. The results of NRI, IDI, and DCA exhibited superior predictive capability and higher clinical use of the nomogram compared with the AJCC staging system.

**Conclusion:**

We successfully constructed a simple and reliable nomogram for OS prediction among PSCC patients receiving RLND, which would be beneficial to clinical trial design, patient counseling, and therapeutic modality selection.

## Graphical abstract

In this study, we successfully constructed a simple and reliable nomogram for survival prediction among patients with penile squamous cell carcinoma. Three clinical variables were integrated into the model, including age, N classification, and log odds of positive lymph nodes. Compared to the AJCC staging system, the established nomogram exhibited superior predictive ability and clinical use.

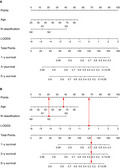

## 1 INTRODUCTION

Penile cancer is an uncommon urologic malignancy, accounting for 0.2% of new cancer cases and 0.2% of cancer deaths worldwide in 2018.^1^ In developed countries, including Europe and the United States, the annual incidence of penile cancer is around 1.0 per 100 000 males.^2^, ^3^ Conversely, it is significantly higher, reaching 2.0‐4.0 per 100 000 males in the developing countries of Africa, Asia, and South America.^4^

Penile squamous cell carcinoma, the most common histological subtype, constitutes 95% of all penile neoplasm.^5^ Due to the rare incidence of PSCC, it is difficult to establish an accurate predictive model for the estimation of prognosis. In 2006, Kattan et al^6^ retrospectively analyzed 175 PSCC patients from Italy and constructed the first nomogram to predict cancer‐specific survival (CSS), clustering lymph node status, tumor thickness, growth pattern, grade, venous and lymphatic embolization, corpora cavernosa infiltration, corpus spongiosum infiltration, and urethral infiltration. However, some of these variables were not routinely provided in the pathological reports, which limited the spread use of this nomogram. In 2009, Zini et al^7^ established a simpler model to predict the CSS of PSCC based on the SEER database, in which the SEER stage and tumor grade were integrated. Subsequently in 2011, Thuret et al^5^ reported that the model relying on the AJCC stage and tumor grade was superior to that using the SEER stage and tumor grade with respect to CSS prediction. Then, Sun et al^8^ proposed a nomogram for prediction of CSS based on the modified tumor and lymph node staging systems. The developed nomogram was then subjected to internal and external validations, showing good discrimination and calibration.

To our knowledge, most of these investigations focused on CSS prediction, and only a few studies concentrated on OS prediction, which was also vitally important for patient counseling and decision making. Additionally, the contemporary cohort of the SEER database was not applied to investigate the OS prediction for PSCC. Recently, lymph node ratio (LNR), defined as the number of positive lymph nodes (NPLN) divided by the number of lymph nodes removed (NLNR), had been demonstrated to be a valuable predictor for recurrence‐free survival,^9^ CSS,^10^ and OS^11^ of PSCC patients. However, it should be noteworthy that the patients with no lymph node involved (LNR = 0) and all lymph nodes involved (LNR = 1) might have different clinical outcomes because of the various lymph nodes yield.^12^, ^13^ Log odds of positive lymph nodes, defined as the logarithmic odds between NPLN and the number of negative lymph nodes, presented prognostic superiority over LNR in some types of cancer.^14^, ^15^, ^16^ In the current study, we constructed a contemporary cohort from the SEER database and devised a nomogram with LODDS for the prediction of OS. Additionally, we evaluated the model performance and clinical use in comparison with the conventional AJCC staging system.

## 2 MATERIALS AND METHODS

### 2.1 Data source

The study population was selected from the SEER database of the National Cancer Institute program (http://www.seer.cancer.gov) from 2004 to 2015. The SEER database covered 28% of the US population and could provide information freely to registered researchers, including basic demographics, primary tumor site, histology, tumor grade, tumor stage, treatment, patient survival data, and so on.^17^ After submitting a SEER Research Data Agreement form, we obtained permission to access the database. We used the software of SEER*Stat (version 8.3.6) to extract the data, and our user name to access the database was 11697‐Nov2018. Our study was exempted from institutional review board approval because of using the de‐identified data in the SEER database.

### 2.2 Study population

**FIGURE 1:**
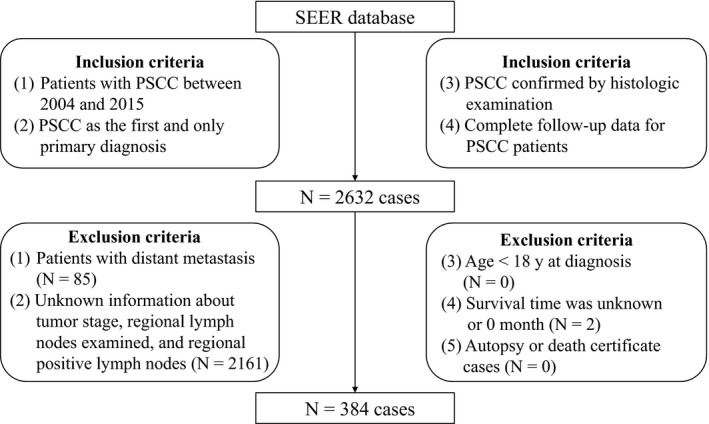
Flow chart of patient selection

The patients with PSCC were identified between 2004 and 2015. Patient eligibility criteria of this study included the following: (a) The patients with PSCC at diagnosis between 2004 and 2015 were enrolled. (b) Penile squamous cell carcinoma was the first and only primary diagnosis. (c) The diagnosis of PSCC was confirmed by histological examination. (d) The patients received RLND. (e) Complete follow‐up data of the PSCC patients could be attained. The exclusion criteria consisted of age <18 years at diagnosis, the presence of distant metastasis, unknown follow‐up data, or unknown information about tumor stage, NPLN, and NLNR. Autopsy and death certificate cases were also excluded. Finally, 384 eligible patients with PSCC were selected from the SEER database. The flowchart of patient selection was presented in Figure 1.

### 2.3 Measurements of variables

For each patient, the demographic and clinical variables were recorded, including age at diagnosis, race (black, white, other), marital status (married, unmarried, unknown), Tumor grade (grade Ⅰ, grade Ⅱ, grade Ⅲ, grade Ⅳ, unknown), T classification (T1, T2, T3, T4), N classification (N0, N1, N2, N3), chemotherapy (yes, no/unknown), radiotherapy (yes, no), regional lymph node examined, regional positive lymph nodes, survival time, and vital status. The patients who received RLND were identified by using the variable “RX Summ‐Scope Reg LN Sur (2003+)” in the SEER database, and the patients with “1 to 3 regional lymph nodes removed” and “4 or more regional lymph nodes removed” were included in the current study. The AJCC Cancer Staging Manual (sixth edition, 2004) was employed to evaluate the tumor stages for the patients identified between 2004 and 2015. The primary outcome of this study was OS, defined as survival from diagnosis of PSCC to death due to any cause. Overall survival was ascertained based on the code “vital status” in the SEER database.

### 2.4 Statistical analysis

Descriptive statistics were carried out to describe the baseline characteristics of the patients. Continuous variables with normal distribution were shown as mean (standard deviation), and non‐normal continuous variables were presented as median (interquartile range [IQR]). Categorical variables were summarized in terms of frequency and percentages. Log odds of positive lymph nodes was calculated through log_e_ [(NLNP + 0.5)/(NLNR‐NLNP + 0.5)].^18^ To avoid singularity, the value of 0.5 was added to numerator and denominator.^18^ Univariate and multivariate Cox regression modeling were subsequently performed to find out the significant prognostic factors of OS. The selection of prognostic factors was performed using a backward stepwise process based on the Bayesian information criterion. Hazard ratios (HRs) and 95% confidence intervals (95% CIs) were used to evaluate the associations between prognostic factors and OS. The proportional hazards assumption of Cox regression modeling was checked with the use of Schoenfeld residuals. No significant association between the Schoenfeld residuals and time was found, which indicated that the developed model was satisfied with the proportional hazards assumption. The nomogram for predicting 1‐, 3‐, and 5‐year OS was subsequently formulated based on the selected prognostic factors.

The original dataset of 384 patients was used to construct the predictive model. Bootstrap validation, a method of internal validation, could reflect the reproducibility of a predictive model and generate bias‐corrected (overfitting‐corrected) performance estimates which were more accurate than the original estimates.^19^, ^20^ In this study, internal validation was performed with the bootstrap resampling method to evaluate the model performance by randomly drawing 1000 samples from the original data set.

Discrimination and calibration, important properties in the evaluation of model performance, were assessed in the current study. C‐index, which depicted the probability of the predicted risk was higher for a random patient having an event than for a random patient not having an event, was applied to evaluate the discriminative ability of the models. The original and optimism‐corrected C‐indexes were both provided. After comparing the predicted probability of events for all possible pairs of patients, C‐index was 0.5 if the model could not discriminate the patients with and without events. Conversely, C‐index was 1 if the probability predicted by the model was always higher for patients with events than those without events.^21^ In this study, calibration plot, the best method to visually compare the accordance between the predicted risk and the actual absolute risk, was presented with bootstrap resampling method.^21^ Calibration plots fall on a 45‐degree diagonal line, reflecting excellent absolute risk estimates. Net reclassification improvement and IDI were usually used to assess and quantify the improvement in risk prediction between the new and old models.^22^ The NRI was based on reclassification tables separately composed of patients with and without events and could quantify the correct reclassification in categories. The NRI could be calculated by adding the percentage of patients with events who were correctly reclassified to the percentage of patients without events who were correctly reclassified.^21^ The IDI could reflect the improvement of sensitivity and specificity, and it also could be viewed as an integrated difference in Youden's indices.^22^ Calculating the IDI required adding the increased probability predicted by the new model compared to the old model for patients with events to the decreased probability predicted by the new model compared to the old model for patients without events.^22^ Net reclassification improvement and IDI were employed to compare the discriminative ability between the new model and the AJCC staging system in the current study. Unlike the sensitivity, specificity, and area under the curve, DCA could be regarded as a method to directly compare benefits and harms which were put on the same scale. DCA plot could represent the model with the greatest net benefits had the highest clinical use, and it was widely used to estimate whether clinical use of diagnostic tests and prediction models would do more good than harm.^23^ In the current study, DCA was conducted to evaluate the clinical use of the nomogram through quantifying the net benefits compared with the AJCC staging system.

Statistical analyses were conducted using R software (version 3.5.3, http://www.r‐project.org/). The significance level was set at *P* < .05, two‐sided.

## 3 RESULTS

### 3.1 Patient baseline characteristics

Between 2004 and 2015, we identified 384 patients with PSCC within the SEER database, and the detailed demographic and clinical characteristics were shown in Table 1. Of the 384 eligible patients, the median age was 61 (IQR: 17). The predominant race was white (84.9%), and most of the patients were married (60.2%). Lymphovascular invasion (LVI) was observed in 72 patients (18.8%). Penile squamous cell carcinoma was grade Ⅰ to Ⅳ in 55 (14.3%), 220 (57.3%), 92 (24.0%), and 2 (0.5%), respectively. T classification was T1 to T4 in 108 (28.1%), 156 (40.6%), 111 (28.9%), and 9 (2.3%), respectively. N classifications were as follows: N0 in 180 (46.9%), N1 in 74 (19.3%), N2 in 84 (21.9%), and N3 in 46 (12.0%), respectively. The median LODDS was −2.43 (IQR: 2.17). With respect to treatment, 97 patients (25.3%) received chemotherapy and 61 patients (15.9%) received radiotherapy.

**TABLE 1:** Demographic and clinical characteristics of patients with SCCP

Variables	All patients (n = 384)
Age, y	61 (17)
Race	
White	326 (84.9%)
Black	35 (9.1%)
Other	23 (6.0%)
Marital status	
Married	231 (60.2%)
Unmarried	138 (35.9%)
Unknown	15 (3.9%)
LVI	
Yes	72 (18.8%)
No	109 (28.4%)
Unknown	203 (52.9%)
Grade	
Grade I	55 (14.3%)
Grade II	220 (57.3%)
Grade III	92 (24.0%)
Grade IV:	2 (0.5%)
Unknown	15 (3.9%)
T classification	
T1	108 (28.1%)
T2	156 (40.6%)
T3	111 (28.9%)
T4	9 (2.3%)
N classification	
N0	180 (46.9%)
N1	74 (19.3%)
N2	84 (21.9%)
N3	46 (12.0%)
LODDS	−2.43 (2.17)
Chemotherapy	
Yes	97 (25.3%)
No/unknown	287 (74.7%)
Radiotherapy	
Yes	61 (15.9%)
No	323 (84.1%)
Follow‐up, mo	
Median (95% CI)	71 (62‐81)

Abbreviations: LODDS, log odds of positive lymph nodes; LVI, lymphovascular invasion.

**FIGURE 2:**
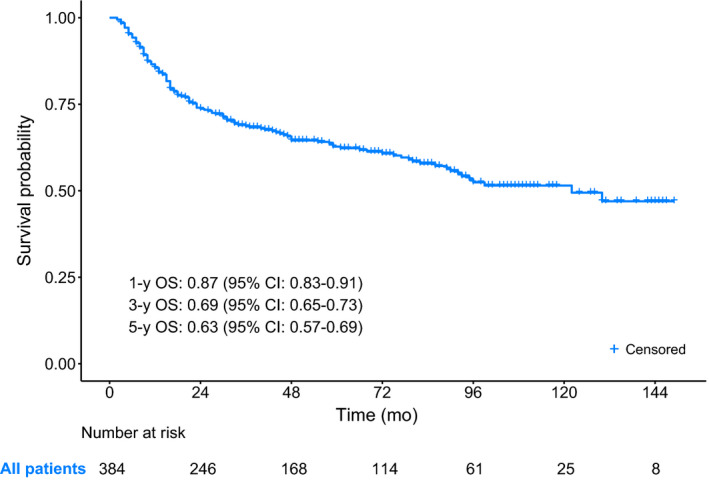
The Kaplan‐Meier curve of OS for PSCC patients with RLND

The median follow‐up time of this cohort was 71 (95% CI: 62‐81) months. By the end of the survey, 147 (38.3%) patients had died, among which 105 (71.4%) died from PSCC, and 42 (28.6%) died from other causes. The Kaplan‐Meier curve of OS for all patients was presented in Figure 2.

### 3.2 Prognostic factors for OS

The Cox proportional hazards regression model was applied to select the significant prognostic factors for OS, and the detailed results were presented in Table 2. Univariate analysis for OS prediction revealed that age (*P* < .001), the presence of LVI (*P* < .001), N classification (*P* < .001), LODDS (*P* < .001), chemotherapy (*P* < .001), and radiotherapy (*P* < .001) achieved statistical significance. After backward variable selection based on the Bayesian information criterion, only three variables remained independent prognostic factors. Age (HR = 1.03, *P* < .001) and LODDS (HR = 1.39, *P* < .001) were independent predictors of OS. The patients with N3 (*P* < .001) and N2 (*P* = .017) had, respectively, 3.36‐ and 1.85‐fold higher risk of suffering death relative to those with N0. The other variables all failed to reach statistical significance.

**TABLE 2:** Univariate and multivariate Cox regression analysis of OS

Variables	Univariate analysis		Multivariate analysis	
	HR (95% CI)	*P*	HR (95% CI)	*P*
Age	1.03 (1.01‐1.04)	<.001	1.03 (1.01‐1.04)	<.001
Race				
White	Ref.			
Black	1.58 (0.95‐2.62)	.079		
Other	0.77 (0.36‐1.64)	.496		
Marital status				
Married	Ref.			
Unmarried	1.21 (0.87‐1.69)	.262		
Unknown	0.49 (0.16‐1.55)	.226		
LVI				
No	Ref.			
Yes	2.38 (1.45‐3.89)	<.001		
Unknown	1.13 (0.73‐1.76)	.577		
Grade				
Grade I	Ref.			
Grade II	0.98 (0.61‐1.59)	.935		
Grade III	1.32 (0.77‐2.24)	.311		
Grade IV:	0.90 (0.12‐6.73)	.921		
Unknown	1.02 (0.41‐2.54)	.958		
T classification				
T1	Ref.			
T2	1.02 (0.67‐1.54)	.929		
T3	1.39 (0.91‐2.12)	.125		
T4	1.30 (0.46‐3.64)	.623		
N classification				
N0	Ref.		Ref.	
N1	2.85 (1.78‐4.56)	<.001	1.49 (0.87‐2.54)	.147
N2	3.49 (2.24‐5.45)	<.001	1.85 (1.12‐3.08)	.017
N3	6.65 (4.15‐10.66)	<.001	3.36 (1.95‐5.77)	<.001
LODDS	1.55 (1.41‐1.70)	<.001	1.39 (1.24‐1.57)	<.001
Chemotherapy				
Yes	Ref.			
No/unknown	0.50 (0.36‐0.70)	<.001		
Radiotherapy				
Yes	Ref.			
No	0.43 (0.30‐0.63)	<.001		

Abbreviations: 95% CI, 95% confidence interval; HR, hazard ratio; LODDS, log odds of positive lymph nodes; LVI, lymphovascular invasion.

### 3.3 Nomogram construction

**FIGURE 3:**
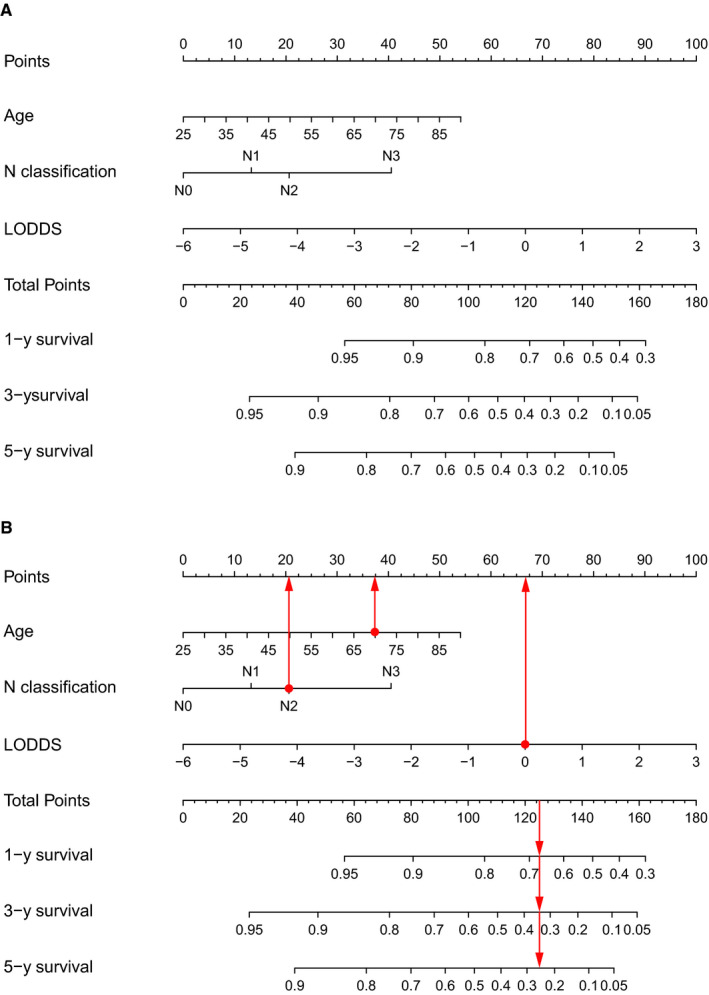
The nomogram for predicting 1‐year, 3‐year, and 5‐year OS of patients with PSCC (A) and an example of using the nomogram (B). Each category of the prognostic variables was assigned a score based on the points scale. After summing up the score of each variable and locating the total score on the total points scale, a line was vertically drawn to the 1‐, 3‐, and 5‐year survival probability scale and estimated survival probability could be obtained

The three independent prognostic factors were used to construct a nomogram to predict 1‐, 3‐, and 5‐year OS. As shown in Figure 3, LODDS made the greatest contribution to prognosis, followed by age. N classification had the least effect on OS.

To help readers to understand how to use this nomogram, an example was presented in Figure 3. A 70‐year‐old patient was diagnosed as PSCC with N2 classification. He underwent regional LND, and LODDS was 0. In Figure 3, vertical lines were drawn from each correct status of the three prognostic factors to the Points scale. Therefore, he had 37 points for the age of 70, 21 points for N2 classification, and 67 points for 0 of LODDS. After summing up each point, a vertical line was drawn from the position of 125 points in the total points scale to the scales of 1‐, 3‐, and 5‐year survival. The total points of 125 corresponded to a 1‐year survival probability of 67%, a 3‐year survival probability of 34%, and a 5‐year survival probability of 25%.

### 3.4 Nomogram performance

**FIGURE 4:**
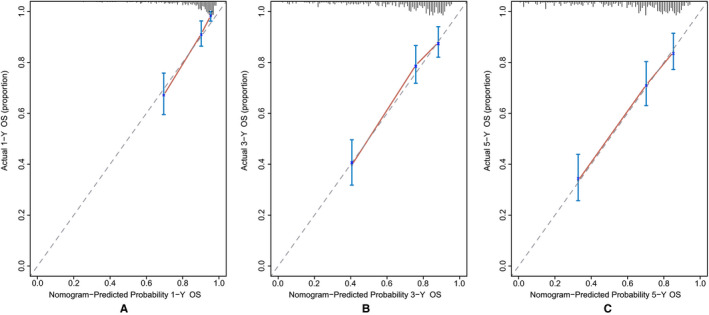
The bias‐corrected calibration plots for predicting 1‐year (A), 3‐year (B), and 5‐year (C) OS of patients with PSCC (Bootstrap procedure with 1000 repetitions). The nomogram‐predicted probability of OS was plotted on the x‐axis, and actual OS was plotted on the y‐axis. The calibration plots could visually represent the relationship between the predicted risk and the actual absolute risk

Discrimination and calibration were both important properties of model performance, which were internally evaluated using bootstrap resampling method in this study. The C‐index of the nomogram (0.746, 95% CI: 0.702‐0.790) was significantly higher than that of the American Joint Committee on Cancer (AJCC) staging system (0.692, 95% CI: 0.646‐0.738, *P* = .020). Across the 1000 bootstrap resamples, the optimism‐corrected C‐index for the nomogram was 0.739 (95% CI: 0.690‐0.784), which was a more accurate and robust performance estimate. In addition, a bootstrapping procedure with 1000 resamples was used to get corrected estimates of predicted and actual values to assess the calibrations of the nomogram. The calibration plots in Figure 4 exhibited excellent accordance between the predicted survival and the actual survival. The NRI values based on bootstrapping for 1‐, 3‐, and 5‐year follow‐up were 0.451 (95% CI: 0.224‐0.696), 0.298 (95% CI: 0.095‐0.532), and 0.369 (95% CI: 0.102‐0.590), respectively. The IDI values for 1‐, 3‐, and 5‐year follow‐up were 0.053 (*P* < .001), 0.076 (*P* < .001), and 0.076 (*P* < .001), respectively. The above results exhibited superior predictive capability of the established nomogram in comparison with the AJCC staging system.

### 3.5 Clinical use

**FIGURE 5:**
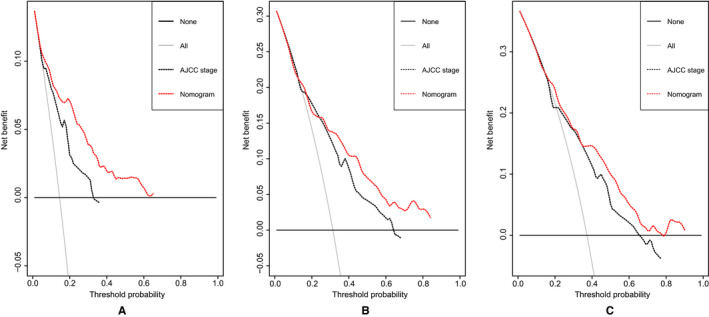
DCA plots of the nomogram and the AJCC staging system for 1‐year (A), 3‐year (B), and 5‐year (C) survival prediction. The horizontal coordinates represented the threshold probability, and the vertical coordinates represented the net benefit rate. The red dash line stood for the DCA of the nomogram, and the black dash line stood for the DCA of the AJCC staging system. The black solid line assumed all patients were alive, and the gray solid line with a negative slope assumed all patients were dead. DCA plot could reflect the model with the greatest net benefits had the highest clinical use

The DCA plots of the nomogram and the AJCC staging system were presented in Figure 5. The nomogram had greater net benefits than the AJCC staging system in the range of threshold probability of 3%‐65% at 12 months, 14%‐17% and 25%‐84% at 36 months, and 15%‐90% at 60 months, respectively. The results indicated higher clinical use of the nomogram relative to the AJCC staging system in OS prediction.

## 4 DISCUSSION

To our knowledge, the prognosis of PSCC patients varies widely, reflecting the clinical and pathological heterogeneity of this disease.^8^ The data from the SEER program showed that 5‐year relative survival rates for penile cancer with localized, regional, and distant stages were 82%, 50%, and 12%, respectively.^24^ Therefore, it is difficult to precisely predict the prognosis of PSCC by using a single predictor, such as the AJCC staging system. The patients with the same tumor stages may have different clinical outcomes due to the ignorance of other significant prognostic factors.^25^ To overcome this limitation, nomograms as reliable tools to quantify risks and calculate the probability of clinical events were introduced, which had been proved to generate more precise prediction than the conventional AJCC staging system in several types of cancers.^26^, ^27^, ^28^ In the current study, the population of PSCC in the SEER database was firstly utilized to develop a nomogram for OS prediction. The established nomogram based on three significant predictors, including age, N classification, and LODDS, presented more accurate assessment and higher clinical use for OS prediction among PSCC patients in comparison with the AJCC staging system.

The involvement of regional lymph nodes was undoubtedly an adverse predictor for survival.^29^ The 5‐year CSS rate of PSCC patients without lymph nodes involvement was 85%‐100%, whereas it was only 16%‐45% for those with lymph nodes involvement.^30^ Based on the AJCC staging system, the 5‐year CSS rate of patients with N1, N2, and N3 classifications were, respectively, 79%‐89%, 7%‐60%, and 0%‐7%.^28^ The current study also corroborated that PSCC patients with higher N classifications suffered worse survival (N3 vs N0, HR = 3.36, *P* < .001; N2 vs N0, HR = 1.85, *P* = .017). The 5‐year survival rate of PSCC patients receiving RLND was 54%, 49%, and 33% for N1, N2, and N3 classifications, respectively.

**FIGURE 6:**
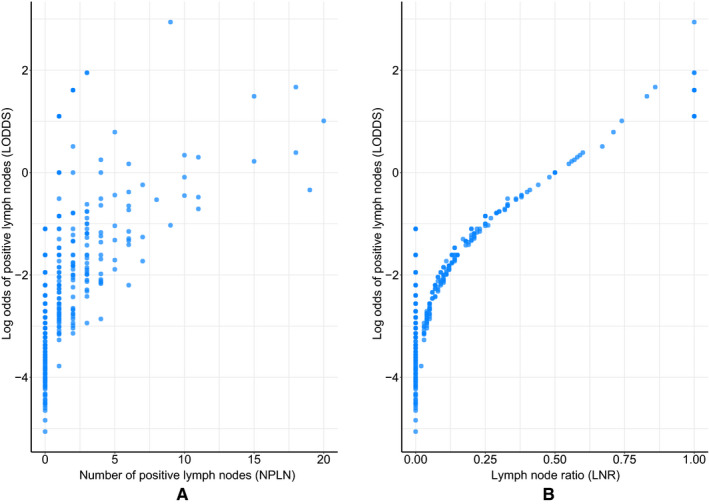
(A) The distribution of NPLN and LODDS. (B) The distribution of LNR and LODDS

To date, investigators have always been seeking a suitable predictor to describe the extent of lymph nodes involvement and precisely evaluate the prognosis of cancer patients. In previous studies, NPLN and LNR, two important aspects of lymph node status, were demonstrated to be independent prognostic factors of clinical outcomes for PSCC.^9^, ^10^, ^11^, ^31^ However, some concerns should be taken into account when incorporating the two variables into predictive models. First, NPLN was an absolute value which could not adequately reflect the scope and extent of RLND. The PSCC patients after RLND with the same NPLN might have different survival due to the different NLNR which was a critical indicator for the quality of RLND.^12^ For patients with lymph nodes involved, more lymph nodes dissection meant a more adequate removal of micrometastatic cancer and a smaller probability of undiscovered positive lymph nodes, indicating a more favorable clinical outcome.^32^ Soodana‐Prakash et al^12^ retrospectively investigated 364 PSCC patients from the National Cancer Database and found that lymph node yield was an independent predictor of OS for PSCC patients with RLND, regardless of N classification. The 5‐year OS of patients with lymph node yield >15 was significantly higher than those with lymph node yield ≤15 (67% vs 49%, *P* = .008). Mao et al^13^ reported that among the PSCC patients with lymph nodes metastasis, more lymph nodes dissection had a beneficial impact on survival. Lymph nodes removed ≥8 were associated with lower all‐cause mortality (HR = 0.48, *P* < .001) and cancer‐specific mortality (HR = 0.42, *P* < .001) than lymph nodes removed <8. Second, the patients with pathological N0 classifications might not be accurately differentiated by using LNR, though, which could provide more information relative to NPLN. Under this circumstance, the patients with declared negative lymph nodes and fewer lymph nodes removed might include some who actually had lymph node metastasis. More lymph nodes dissection increased the chance of correct staging, which further increased the likelihood of appropriate treatment selection, and therefore, improved the survival of PSCC patients who were understaged.^12^ A study by Li et al revealed that lymph nodes removed ≥16 could significantly improve the CSS of PSCC patients with pathological N0 classifications.^10^ A multicenter study of China exhibited that the increased count of lymph node examined was significantly associated with classification migration from N0 classification to N1 and N2 classifications (OR: 1.012, *P* < .001) and OS improvements (N0 classification, HR: 0.981, *P* < .001; N1 and N2 classifications, HR: 0.984, *P* < .001) for non–small‐cell lung cancer, which was also validated by using the SEER database.^32^ To address the limitations of NPLN and LNR, LODDS was introduced in the current study, which could provide more information than NPLN and better discriminate PSCC patients with no and all lymph nodes involvement than LNR.^14^ As shown in Figure 6, LODDS could better differentiate the patients with the same NPLN and LNR. Additionally, we also tried to construct predictive models by using NPLN, LNR, and LODDS, respectively. The C‐index of the predictive model with LODDS (0.745, 95% CI: 0.701‐0.789) was higher than those of the models with NPLN (0.715, 95% CI: 0.678‐0.766) and LNR (0.737, 95% CI: 0.692‐0.782), though statistical significance was not achieved.

Until now, investigations regarding predictive models for OS prediction of PSSC have been rare. Necchi et al^33^ identified 689 PSCC patients receiving RLND from 1980 to 2017 and found age (*P* < .001), LNR (*P* < .001), N classification (*P* < .001), and received neoadjuvant chemotherapy (*P* = .013) were significantly associated with OS. A nomogram was then proposed with good discriminative capability (c‐index: 0.75), clustering age, LNR, N classification, receive neoadjuvant chemotherapy, pelvic LND, bilateral LND, and adjuvant chemotherapy. Of note, the eligible patients in this study were identified in a 37‐year span, which could not be regarded as a representation of contemporary patients, and this limitation might decrease the reliability and extrapolation capability of the model. In addition, as clinicians, we commonly paid more attention to the clinical use of the new model, especially in comparison with the conventional AJCC staging system. Unfortunately, this study did not provide the information which we were interested in.

The current study had its own merits. Based on the population‐based SEER database, a simple nomogram for PSCC with only three variables was established and validated, showing superior predictive ability and higher clinical use compared with the AJCC staging system. Unlikely other complicated models, the involving variables in our model could be conveniently attained from the routine pathological reports, which also represented a strength. As we know, the predictive model should be accuracy and parsimony. In clinical practice, a complex tool, especially with a large number of predictors, probably could not be implemented because of time considerations and the potential unavailability of predictors.^7^ Additionally, LODDS as an independent predictor was firstly incorporated into a nomogram to predict the OS of PSCC patients, exhibiting a potential prognostic superiority relative to other lymph node staging systems.

Though some progress has been made, a few limitations should be noteworthy. First of all, the main drawback of our study is the retrospective nature, and the bias in the process of patient selection cannot be avoided. Second, some variables associated with the prognosis of PSCC are not recorded in detail in the SEER database, such as therapy modality. This limitation prevents us from incorporating more valuable predictors into the current model to improve predictive accuracy. Third, similar to the previous study, the independent external validation was not performed due to the rare incidence in our single hospital, which should be furtherly carried out to increase the reliability of the predictive model.

## 5 CONCLUSIONS

In conclusion, to the best of our knowledge, a simple nomogram for OS prediction was firstly established by using a contemporary cohort of PSCC patients from the SEER database, in which only three variables, including age, N classification, and LODDS, were integrated. Despite its limitations, our model represented superior predictive capability and higher clinical use in comparison with the conventional AJCC staging system.

## CONFLICT OF INTEREST

The authors declare no conflicts of interest.

## AUTHOR CONTRIBUTIONS

Wenwen Zheng and Kangqi Li participated in study design. Weiwei Zhu, Yuexia Ding, Qingna Wu, and Qiling Tang were responsible for data collection and analysis. Wenwen Zheng was involved in drafting the manuscript. Congxiao Lu, Quan Zhao, Shengqiang Yu and Chenyu Guo revised the manuscript. All authors read and approved the final manuscript.

## Data Availability

The raw data supporting the conclusions of this manuscript will be made available by the authors, without undue reservation, to any qualified researcher.
